# Definition and improvement of the concept and tools of a psychosocial intervention program for parents in pediatric oncology: a mixed-methods feasibility study conducted with parents and healthcare professionals

**DOI:** 10.1186/s40814-019-0407-8

**Published:** 2019-02-06

**Authors:** David Ogez, Claude-Julie Bourque, Katherine Péloquin, Rebeca Ribeiro, Laurence Bertout, Daniel Curnier, Simon Drouin, Caroline Laverdière, Valérie Marcil, Émélie Rondeau, Daniel Sinnett, Serge Sultan

**Affiliations:** 1 Sainte-Justine University Health Center, Chaussée de la Côte-Sainte-Catherine, 3175, Montréal, Québec H3T 1C5 Canada; 2 Department of Psychology, Université de Montréal, Québec, Canada; 3 Department of Pediatrics, Université de Montréal, Québec, Canada; 4 Department of Kinesiology, Université de Montréal, Québec, Canada; 5 Department of Nutrition, Université de Montréal, Québec, Canada

**Keywords:** Pediatric cancer, Parents, Supportive care, Psychosocial intervention, Intervention development, Mixed methods

## Abstract

**Background:**

Studies have shown that supporting parents in pediatric oncology reduces family distress following a cancer diagnosis. Manualized programs for parents have therefore been developed to reduce family distress. However, these programs have limitations that need to be improved, such as better defining programs’ procedures, developing interventions focusing on parents’ conjugal relationship, conducting rigorous evaluations of implementation, and proposing adaptations to various cultural dimensions. According to the Obesity-Related Behavioral Intervention Trials (ORBIT) model for the development of behavioral intervention, we improved these limitations and developed TAKING BACK CONTROL TOGETHER, a six in-person intervention sessions to support parents of children with cancer by taking the active components of two programs: Bright IDEAS and SCCIP. Referring to the redesign phase of the ORBIT model, this study aims to refine the definition of this program’s design by interviewing parents and healthcare professionals.

**Methods:**

In order to refine the program, we used a sequential mixed-methods study. Parents and healthcare professionals first completed questionnaires assessing the program, and then discussed its limitations, benefits, and areas for improvement in group and/or individual interviews. We performed a descriptive thematic content analysis of the qualitative data from the open-ended questions (questionnaires and interviews) with NVivo 11 to categorize recommendations for the program refinement.

**Results:**

The results showed that components seemed pertinent to final users. The main areas needing improvement were the level of complexity and understandability of the parent manual, the possibility to choose the place and time of the intervention, and the lack of ethnic/cultural diversity. Changes to the program were made accordingly.

**Conclusions:**

It is necessary to include end-users when developing complex intervention programs designed for vulnerable populations and sensitive clinical contexts. Following the present refinement, we now have a treatment package, which is safe and acceptable for the target population and has a better chance of yielding a clinically significant benefit for users in a future pilot study.

## Background

Distress in parents who are confronted with their child’s cancer diagnosis is characterized by a wide range of symptoms such as stress, uncertainty, loss of control, anxiety, depression, and traumatic symptoms [1–3]. According to studies, this distress is related to difficulties in parents’ ability to adapt to the oncological situation and being overwhelmed in the management of daily life [4, 5]. Studies have also shown that mixed parental distress, consisting of anxiety, depression and traumatic stress, is associated with long-term distress and adaptive difficulties in the child himself/herself, as well as impaired school functioning [6–8]. In addition to each parent’s distress, studies have also highlighted the specificities of conjugal distress in pediatric oncology [9, 10]. Parents’ intimacy, sexuality, time, and activities were negatively influenced by the child’s cancer. Importantly, it has been shown that spousal support on the current emotional and conjugal adjustment of each spouse improves the couples’ functioning in both the short and long term [11]. These findings are in line with the reduction in psychological distress and conjugal conflict, and a greater satisfaction with marital life observed among both fathers and mothers who perceived increased support from their spouse [12].

These studies suggest that it is crucial to support parents, treat their distress as early as possible, and promote unity and marital adjustment to maximize family resilience to childhood cancer. In order to evaluate the psychosocial support offered to parents, we conducted a systematic review to identify evidenced-based manualized programs that had been developed, and how they were designed and implemented in pediatric oncology clinics [13]. We identified 11 manualized intervention programs designed to support parents following their child’s cancer diagnosis. These programs followed various models of change including cognitive behavioral therapy (CBT), psychoeducation, and systemic therapy. An in-depth analysis of existing intervention programs, their structure, and tools, as well as their hypotheses of change has highlighted multiple limitations in their definition of a design that needs improvement. On the one hand, a number of programs did not consider extant components with documented implementation and effects. Other programs did not always accurately document how targets and models of change were selected. On the other hand, several programs lacked a design refinement as well as pre-test and test phases that would have improved their acceptability, feasibility, and effectiveness. Finally, the programs were mostly defined for a specific population and were therefore not applicable to individuals from different cultures. This review’s results are available in another publication [13].

Based on this review, we retained two programs, Bright IDEAS and SCCIP, which have the best effects on their primary outcomes and are the only ones recommended by the US National Cancer Institute (NCI) [14, 15]. Bright IDEAS is an intervention program based on problem-solving skills training (PSST). Although Bright IDEAS has excellent NCI scores on dissemination (5/5) and research integrity (4.4/5), this program still bears limitations. Firstly, because it is only offered to mothers and administered individually, it received a limited impact score (2/5). Secondly, because it is relatively burdensome and somewhat repetitive (eight in-person sessions), the dropout rate was high (42%) [16]. SCCIP is based on the principles of CBT and systemic therapy. SCCIP relies on different activities based on family interventions aiming to improve coping and marital/intrafamily communication. For instance, it relies on multiple family discussion groups which suggest conducting a group discussion to clarify parental functions. This activity’s strength is its family-oriented approach which helps families cope with cancer [17, 18]. SCCIP has received fair NCI scores on research integrity (3.6/5) and dissemination (4/5), but a lower score on impact (1.3/5) [19]. The manual remains largely general with regards to the complexity required for communicational interventions in the family and lacks important details about the specific interventions to be carried out. Thus, it is difficult for professionals to use the interventions prescribed by this program in a systematic way. Of note, Bright IDEAS was developed in two languages (English and Spanish), whereas SCCIP was only developed in English. Like the vast majority (8/11) of available interventions, these programs were developed for a North American population which raises questions about these programs’ transferability to individuals who are from different cultural backgrounds or who do not speak English and/or Spanish [13].

Based on these observations, we addressed these programs’ limitations by combining their most effective procedures and translating them into a program better suited for a French-speaking population in pediatric oncology [20]. In order to translate and correctly articulate these programs, members of our team were trained in both programs and met with the respective authors. The resulting program was therefore a new integrative intervention offering an active approach to meet parental needs. Several frameworks have been formalized for optimizing interventions [21–23]. Among these, Obesity-Related Behavioral Intervention Trials (ORBIT) is a complete evaluation model for intervention programs which explicitly describes the preliminary stages of program development [24]. This model consists of four phases: I, program definition phase; II, preliminary tests; III, efficacy studies; and IV, effectiveness studies. Phase I, in which the program is defined, consists of two stages: Ia, the definition, and Ib, the refinement of the design. With regard to the definition of the new program, TAKING BACK CONTROL TOGETHER, we followed the ORBIT phase I recommendations: 1, definition of the target population; 2, selection of the program’s primary and secondary intervention targets of; 3, justification for the choice of models of change and their translation into interventional procedures; and 4, development of a coherent program comprising these procedures [25]. In order to standardize the administration of this intervention, we also created a manual for practitioners and parents, consistent with standard practice in behavioral sciences [26].

**Fig. 1:**
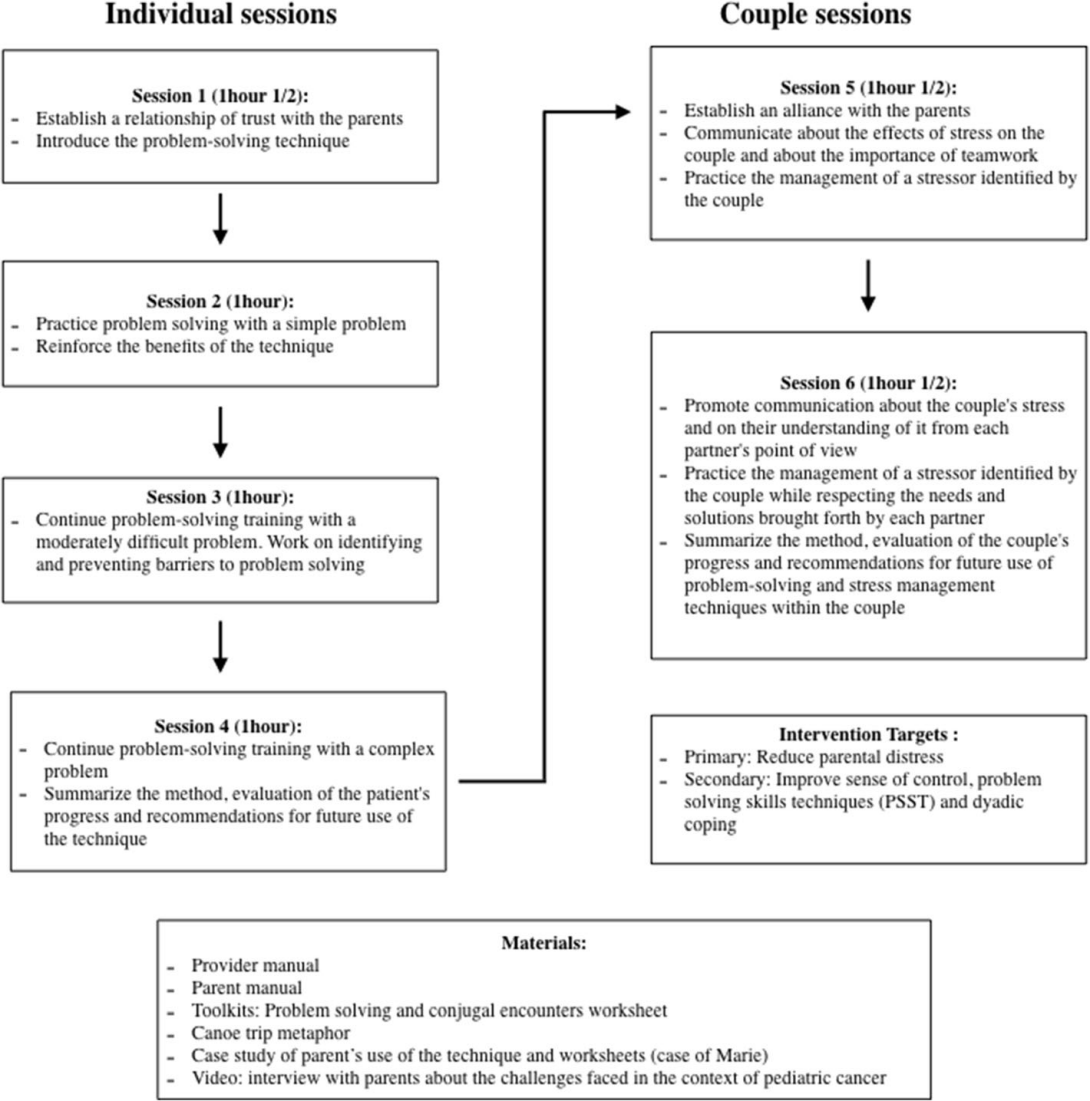
Initial design of the intervention TAKING BACK CONTROL TOGETHER

TAKING BACK CONTROL TOGETHER aims to strengthen parents’ sense of control and PSST (heterosexual and homosexual couples) and focus on dyadic coping to prevent distress. This program is based on cognitive behavioral and systemic theories. It includes six sessions: four individual sessions, offered to each parent, and two couple sessions (see Fig. 1). The individual sessions focus on PSST, as well as acquiring, developing, and maintaining simple problem-solving skills to meet the needs of families facing childhood cancer. PSST includes six stages: 1—problem selection; 2—problem definition and operationalization; 3—generation of possible solutions; 4—decision-making; 5—solution implementation; and 6—effectiveness evaluation [27]. These sessions take place at the hospital during the child’s treatment. They can also be offered to single-parent families. Both couple sessions are based on CBT and systemic therapy. They aim to enhance parents’ communication and resilience by improving their ability to manage real difficulties associated with childhood cancer together [9, 28]. Couple sessions are provided either at the hospital or at the parents’ residence according to the parents’ preference. In blended families, each parent can participate in the program with their new partner. A manual for healthcare professionals (provider manual) provides specific instructions for each intervention to be used in every program session. Furthermore, the provider manual offers numerous transcripts examples to convey the information to parents adequately and in a standardized manner. A manual for parents includes PSST toolkits for individual and couple sessions, as well as strategies related to communication and dyadic coping. Three illustrations are also available: (1) a video about a canoe trip disturbed by bad weather to illustrate problem-solving steps; (2) a written clinical vignette illustrating an example of how a problem related to a child’s cancer is solved by the parents; and (3) a video in which parents of children with cancer discuss the challenges they face in their conjugal relationship. A complete description of this intervention’s design definition is available in a study report [20].

Following the intervention’s definition, design refinement is an essential step in program development that is also necessary to document. The intervention’s design refinement (ORBIT phase Ib) aims to identify essential treatment components, determine aspects related to its administration (such as its frequency and duration for example), and improve its strength and efficiency [24]. The milestones to achieve in order to move to the following step (preliminary testing of the ORBIT phase II) is to have established the treatment package’s essential components, to ensure that it is safe and acceptable for the target population, and that it is likely to produce a clinically significant positive effect on the target. During these preliminary tests, we will conduct a pilot test which will assess the new program’s acceptability and feasibility. Following these preliminary tests, efficacy (ORBIT phase III) and effectiveness (ORBIT phase IV) studies could be conducted.

The study’s main objective was to refine TAKING BACK CONTROL TOGETHER following interviews with end-users: pediatric oncology healthcare professionals and parents of children with cancer. Specifically, this study aimed to (1) evaluate and (2) provide suggestions to improve the program’s relevance, acceptability, material, practical implementation, and technique procedures [29].

## Methods

### Procedures and participants

#### Participants

We recruited a convenience sample of healthcare professionals and parents through internal email and online social media, respectively. A post was published on the Leucan Facebook page, a community support organization for children with cancer. The post invited parents to participate in the study and outlined the study’s inclusion criteria. Such recruitment procedure is frequent with populations who are difficult to reach and allows participants to take part in the study on a voluntary basis [30].

To be included in this study, healthcare professionals had to be members of the hematology-oncology staff and have worked for more than a year in the pediatric field. We included one participant from each of the following practices: medical, nursing, social, psychological, physiotherapy, and volunteering team. Parents included in the study had to have a child in remission for at least 6 months and the ability to speak French. The decision to gather experiences from parents whose children are in remission is associated with the desire to evaluate the program’s components with participants who are somewhat at some distance of their experience with pediatric cancer. By having more perspective on the disease, they are able to share the experience they have lived at different times, from diagnosis to remission. Information power was reached through pertinence of selected participants and not by the importance of their number [31].

#### Procedures

A sequential mixed-method design was used to assess the program. Mixed questionnaires with closed- and open-ended questions as well as directive interviews (individual, couple, and group) were employed. First, we met healthcare professionals, then we met the parents. Meeting healthcare professionals first and parents second is a decision supported by the criterion of methodological coherence and by clinical evidence. We wanted to have clinicians’ opinion to avoid any inconsistency with the care of parents.

Healthcare professionals included in this study were invited to a group meeting involving three steps. The first step was a 30-min information session about the project. Beforehand, they had received documentation on the program to become acquainted with it. The documentation consisted in provider and parent manuals as well as intervention tools. As a second step, they were invited to complete an evaluation questionnaire in paper format (described below). The third step was to take part in a focus group investigating the questionnaire’s main themes and focusing on elements that had been negatively evaluated in step two, i.e., the providers were invited to discuss these elements and to suggest ways to improve them. This focus group was conducted by two researchers (DO, RR), audio recorded and transcribed.

After completing the focus group with the professionals, the same two researchers (DO, RR) met with the parents. Given the difficulty of finding a time that suited all parents for a group interview, only individual or couple sessions were conducted. The procedure of these meetings was as follows. Firstly, participating parents were contacted by phone to introduce the intervention and verify the inclusion criteria. Secondly, a brief description of the study, the parent manual, intervention tools, and the parent questionnaire were sent to them by email or surface mail. After having read the documents, parents were asked to complete an evaluation questionnaire in PDF format on their computer or tablet (described below). Thirdly, 1 week after the initial contact and the description of the program, interviews with parents were conducted to discuss both the questionnaire’s results and the comments made earlier by professionals. We also asked for their input as to how to improve the intervention accordingly. When parents were unable to come to the hospital, a 1-h meeting following the same structure was offered by phone. These sessions were audio recorded and transcribed. Finally, after data analysis was completed, the intervention program’s manuals and tools were improved following the participants’ suggestions.

Participating parents were compensated for their participation. Healthcare professionals did not want to receive financial compensation for their participation. Therefore, a donation of 30$ was made to an association of their choice. The CHU Sainte-Justine Ethics Committee approved this project.

#### Measures

Socio-demographic information was collected for this study. For healthcare professionals, we collected occupation, age, and years of experience. For parents, we collected marital status, number of children, occupation, work status, and the date of diagnosis.

The research team also created two questionnaires by adapting questions used in previous studies [32, 33]. The questionnaire for healthcare professionals included 50 items grouped into four categories to evaluate the program’s relevance and acceptability (6 items, e.g., “the program is adequate to deal with difficulties experienced during childhood cancer”), the program material (5 items each about provider and parents’ manual as well as intervention tools, e.g., “the manual provides sufficient information to parents, program tools are easy to use”), the program’s practical implementation (8 items, e.g., “you could invest in the six-week program”), and the expected effects of its procedures (21 items, e.g., “the program would have taught you something useful”).

Responses were provided on a 6-level scale, from 0 “strongly disagree” to 5 “strongly agree.” For each topic, space was available to allow participants to write comments.

Parents’ questionnaire was the same as the one completed by healthcare professionals with the exception that it did not contain any questions about the provider manual and included more questions about the program’s expected effects. Parents’ questionnaire included 49 items grouped into four categories: the program’s relevance and acceptability (6 items), the program’s material (5 items about parents’ manual and 5 about intervention tools), the program’s practical implementation (8 items) and the expected effects of its procedures (23 multiple choice items and two open-ended items, e.g., “how do you rate problem solving as a relevant skill to help parents of children with cancer?”). The response format was the same as for professionals.

### Analyses

Quantitative data were analyzed using descriptive statistics (frequencies) in order to identify and prioritize the critical elements to be discussed in the interviews. Qualitative data from the open-ended questions (questionnaires) and transcriptions (interviews) were processed and encoded using NVivo 11 software by analysts who received appropriate training specifically for this project [34]. An emergent thematic analysis method was used, and the transcript coding process followed these steps: (1) outlining of the significant critical categories for descriptive analysis; (2) encoding of the transcript by two authors separately (DO, RR); (3) completion of two meetings to discuss and evaluate the inter-rater agreement and the themes selections between the authors by consensus (DO, CJB, RR); (4) recoding of the transcript following each meeting, based on established guidelines and code descriptions; (5) development of the hierarchy of the three central themes (parents’ experience, evaluation of the program, suggestions for improvement); (6) reduction and synthesis of the data for each theme; (7) a preliminary drafting of the results incorporating quotes that had been transcribed without the markers of spoken language to allow for a more fluid reading [35].

Quantitative results relate to the average scores of the main topics assessed by questionnaires (total score for each item is 5). They are summarized in a table and presented in context with the qualitative results. The first section of the result from qualitative data is presented in a narrative form. Participants are identified using alphanumeric codes (P1 to P6 for parents and H1 to H6 for healthcare professionals) [36]. Short quotes were embedded in the text in quotation marks, and longer quotes are presented in a distinct paragraph.

## Results

### Participants

Six of the 22 healthcare professionals who were invited agreed to participate in this study. In order to have a sample that adequately reflects the diversity of healthcare professionals in pediatric oncology, the researchers ensured the sample’s internal diversity by recruiting a representative from various professions: a doctor (H1), a psychologist (H2), a social worker (H3), a nurse (H4), a physiotherapist (H5), and a librarian of a pediatric oncology foundation (H6). These professionals were in average 48 ± 9.5 years old and had 20.3 ± 8.5 years of experience.

Twelve parents responded to the invitation to participate in this study. Of these responses, six parents—two married heterosexual couples (P1, P2; P3, P4) and two mothers; one married (P5) and one divorced (P6)—who had lived through their child’s cancer experience agreed to participate and were included in the study. Both couples were interviewed at the hospital and the two mothers participated by phone, as they were unable to come to the hospital for these appointments. Parents reported an average age of 45.3 ± 8.4 years. Concerning the parents’ professional activities, there were two biologists, one nurse, one specialized educator, one electrician, and one without employment. Parents who were not included in this study (six people) indicated that they lived too far away from the hospital to participate in the research interviews, were no longer interested following the phone call, or did not respond following the mailing of the documents.

### Qualitative analysis of parents’ experience

Both healthcare professionals and parents began by talking about the parents’ experience. In summary, parents reported having experienced a significant shock at the time of the diagnosis, which caused emotional overload and a loss of control. The complexity and quantity of information received by parents also increase this overload and may cause a feeling of uncertainty. In response to this emotional difficulty, some parents reported having used problem- (or conflict-) solving strategies on their own or within their couple. The importance of control in the search for information or in discussions with specialists was also pointed out. In addition, aspects of marital/family coping were raised, such as the importance of maintaining unity and intimacy within the couple, communicating well and sustaining family routines. Time also allows one to adapt to this situation.

### Program evaluation

Responses to questionnaires assessing the program showed average scores for healthcare professionals and parents (see Table 1). As a conclusion to these quantitative results, several topics were raised to be discussed in focus groups and interviews, including the program’s relevance and acceptability, the usefulness and the writing styles of both manuals, the program’s practical implementation, and the expected effects of the procedures on parents.

**Table 1:** Average scores of the responses to the questionnaires (total score out of 5)

Theme	Health professionals	Parents
1—Program relevance and acceptability	3.8	3.5
2—Program material	4.1	4.7
Provider manual		
- Usefulness	4.5	Not rated
- Writing style	4.3	Not rated
Parent manual		
- Usefulness	4.1	4.6
- Understanding	2.8	5
Tools kit		
- Worksheets	4.3	4.6
- Illustrations and case study	4.8	4.6
3—Practical implementation	3.2	3.9
Location of the intervention		
- Hospital	3.3	4.5
- At parents’ home	3.3	4
Time of the intervention	3.5	3.8
Number of sessions	3	3.6
Tasks to complete at home	2.8	3.8
4—Program procedures	4.5	3.5
Improve sense of control	4.3	3.2
Improve problem-solving skills	4.8	3.8
Improve dyadic coping	4.3	3.6

#### Program relevance and acceptability

Healthcare professionals and parents positively evaluated the program’s relevance and acceptability (*M* = 3.8/5 and 3/5 respectively). Firstly, several benefits were observed among professionals and parents. According to all health professionals, this program will provide parents with psychological support and help them better manage the emotions related to their child’s medical situation. It will also be beneficial in supporting parents in the daily management of their sick child’s situation; in helping them cope with treatments, and in planning and organizing the child’s return home. One healthcare professional (H4) believes that the program will also contribute to the management of resources because it “can be connected with the standard social services.” This perception is shared with parents, who appreciated the fact that the program aims to help them take back control, especially in the management of the practical difficulties they face (work interruptions, daily tasks, child care). One couple (P3, P4) expected benefits even following the illness and their return to work as well as the possibility of “later generalizing their newly acquired skills.” Another benefit highlighted by parents is the management of emotions, including sadness, which the program promotes targets. One mother (P6) pointed out that “the disease brings out despair and the intervention helps manage despair.” She added that for her, the sense of control was associated with the fact that parents must be available for their child and must try to provide them with the best possible environment. Another mother (P2) saw the program as an opportunity for necessary and beneficial downtime:

P2: “Being able to sit down and have a tool to communicate while remaining cool-headed because it is always intense…it can have positive effects.”

Parents also confirmed the choice of the target audience. Two couples (P1, P2) (P3, P4) supported the idea that this program should be offered to parents who are experiencing the most trouble (in distress), separated couples and single-parent families. One father (P3) also noted that pediatric cancer spares no particular group:

P3: “The disease affects everyone, millionaires, and disadvantaged people who already have problems.”

On the other hand, healthcare professionals reported that a potential difficulty with the problem-solving technique was the way it would be applied by parents. For most of them, the model of the program is not flexible or adaptable enough to the realities of some parents:

H5: “Should we consider a shorter and longer program to adapt to the realities and problems encountered? Moreover, what if [the parents are] in denial of the problem or problems? How do we avoid turning it into a psychotherapy session? I think a lot will depend on where the parents were pre-diagnosis.”

Additionally, according to the healthcare professionals (H4, H5, H6), it is better to avoid using words and expressions that are too related to school vocabulary in this program such as “homework” when speaking of the prescription of tasks to complete at home, the term “resolution” in the technique’s title used in the individual sessions or the word “problem,” as these terms may have a negative connotation and influence the course of the sessions as well as the patients’ mood. This vision was not shared by all parents; only one mother (P4) argued that the program must also address how to talk to parents:

P4: “The word calm, for example, you know when someone is angry and then you ask: are you calm? … you are pffff…”

#### Program material

In general, the usefulness of the parent manual was rated very positively by healthcare professionals (*M* = 4.1/5) and by parents (*M* = 4.6/5). On the other hand, the writing style of the parent manual was the subject of several remarks from healthcare professionals (*M* = 2.8/5), while it was rated very positively by the parents (*M* = 5/5). Indeed, healthcare professionals indicated that some parents in the target population could have difficulties understanding and dealing with the vocabulary, the writing style, and the amount of reading of this manual.

H1: “The paragraphs were too long. Look at, for example, the stuff on LaPresse or the BBC, paragraphs are three lines, four lines, maximum. If they are stressed individuals, you cannot have long paragraphs like that.”

H4: “Parents have many things to read with the diagnosis, with the drugs and the treatments...You read about the diagnosis, you read about the medications, you read about the care that is the thing.”

The majority of interviewed parents approved of the program’s manualized aspect and the utilization of worksheets that allowed them to expand their learning and knowledge of the program outside of the in-person sessions, as well as to have the choice to participate as a couple or individually. One mother added that everyone could use the manual when the right time comes:

P4: “When the person is alone, the brain is relaxed, while the little one is sleeping in his room, it can be good to see this.”

Moreover, according to several healthcare professionals, the cognitive impact related to the level of distress is also to be taken into account when writing the manual. One healthcare professional (H4) argued that the presentation should remain simple because, for at least the first 4 weeks following the diagnosis, the parents are in “survival mode.” Some parents also expressed doubts about their ability to learn in this context:

P1: “Of course there could be parents who would not be able to read this, that is for sure.”

P6: “When we learn about our child’s cancer, we become less intelligent (for a while).”

#### Practical implementation

Healthcare professionals and parents reported different judgments on the practical modalities of the intervention program. Healthcare professionals were satisfied with the conduct of program’s sessions at the hospital (*M* = 3.3/5) and at home (*M* = 3.3/5). Parents highlighted several advantages concerning this topic. They generally appreciated having the flexibility to choose the location of the intervention program either at the hospital (*M* = 4.5/5) or at home (*M* = 4/5) for the individual and couple interviews. The overload caused by the medical situation is already very demanding, and one mother (P2) mentioned that offering the opportunity to hold couple meetings at home in the evening can make a difference because “it’s one less trip for people living outside of the city.” With respect to individual sessions, one mother (P4) indicated the benefits she sees in having them at the hospital:

P4: “The hours and days at the hospital get long, so there is a lot of time for the meetings.”

The proposed time to start the intervention—approximately 4 weeks post-diagnosis and adjustable according to the parents’ needs—was satisfactory for healthcare professionals (*M* = 3/5) and positively perceived by parents (*M* = 4/5). According to one mother (P5), it is appropriate to allow a minimum period of 3 weeks following the diagnosis, which allows people to “breathe a little, between visits to the hospital and very intense worrying.” Healthcare professionals (H4, H5) stressed the importance of being flexible in planning the program and in offering choices to parents because for some of them “it is intrusive, invasive.” Because each family is different, parents do not all react the same way or at the same pace:

H5: “There may be families who take action right after the diagnosis, and maybe this kind of program could be beneficial very early on. However, others may be completely frozen following the diagnosis, so it takes more time before we can accompany them to move forward.”

Conversely, healthcare professionals reported more reservations than the majority of parents on this topic. They mentioned the parents’ lack of availability to participate in the program. According to some of them, parents may decline to participate in order to focus all of their available time on their sick child, their marital relationship, and on other family members. One father (P1) also shared this point of view:

P1: “Basically, the way I see it is that one of the two should be available... I do not think it is realistic that it be the father and the mother actually.”

Another concern expressed was regarding holding meetings at home. Contrary to parents (P1, P2) who approved of the importance of giving parents a choice as to the location of the intervention, healthcare professionals expressed doubts regarding the relevance of interventions at the parents’ home. Some believe that the presence of a healthcare professional could be intrusive in the family dynamics, could increase the parents’ overload, and could cause them to feel evaluated, judged, or forced merely to tidy up their home. One father (P1) also pointed out that there might be parents “who really do not want you to go to their home...”

In general, the number of sessions was deemed satisfactory (*M* = 3/5 for healthcare professionals and *M* = 3.6 /5 for parents). However, it was considered high for one of the couples that were interviewed (P1, P2), who thinks that it should be adapted according to availability and need:

P1: “It depends on the person’s needs or level of disruption. You know, if you can tell that the person has never lost control, you do not need six sessions. Then there are some who have never had that kind of control...and somehow it could serve them.”

Finally, participants mentioned no limitations concerning the program’s components. The prescription of tasks to complete at home, however, was the subject of differing opinions: too demanding according to professionals (*M* = 2.8/5) but relevant according to parents (*M* = 3.8/5), especially to one mother:

H3: “At times, parents already have too much to learn: learning how to use the central venous catheter, learning how to use the feeding tubes, learning so many new things that they need to do at home.”

P2: “The homework is good. I think it is a good workload (…) It circles in your head; you figure out what issue you would like to work on. So, I don’t think it is too much because you do not actually need to write it all. We will think about it if we receive the intervention.”

#### Program procedures

All participants felt that the program’s individual component is relevant and very much in line with the goal that had been defined, i.e., to strengthen parents’ sense of control. Participants evaluated the individual sessions positively. They help to improve the sense of control (for healthcare professionals, *M* = 4.3/5; for parents, *M* = 3.2/5) and PSST (for healthcare professionals, *M* = 4.8/5; for parents, *M* = 3.8/5). For healthcare professionals (H4, H5), the individual sessions are important because they make it possible to present the approach thoroughly. For parents, these meetings allow them to speak more openly and help them express anxiety and regulate their emotions. One mother (P2) mentioned that the parents’ well-being depends on the child’s medical situation and that they “must learn to live with cancer.” Another mother (P5) suggested that these sessions will allow them to take back control over emotions and thus be better equipped to support their child:

P5: “To better manage, you cannot talk about acceptance because it is hard to accept. We should instead take back control over our functioning.”

Participants also considered the two couple sessions relevant. They believed that these sessions strengthen the couple’s unity (for healthcare professionals, *M* = 4.3/5, for parents, *M* = 3.6/5). Many parents believed that marital communication will be improved following these sessions, which will help them in the management of couple crises and misunderstandings. One mother (P5) stated that as soon as the diagnosis is announced, parents need to be able to communicate and cooperate together and that the program can enrich the relationship and optimize their ability to “communicate without judgment.” One mother (P6) also mentioned that it is essential to prioritize the couple because the disease takes up considerable space and there are few moments when the couple can get together to talk because, in her point of view, “it is important to have a joint approach.” One couple (P3, P4) mentioned that most of the time, one parent is at the hospital and the other at home, but that they still need to meet to discuss the rest of the family, who are often neglected in this context, and that the program helps create a “moment together.”

Parents also approved being assigned tasks to complete at home, on the condition that the tasks are not imposed and do not add to the overload. As one mother emphasized (P5), these tasks should be completed “only if the person wants to do them, if they feel energized.”

#### Program provider’s attitude

The program provider’s attitude was discussed during focus groups and interviews. According to healthcare professionals, it is essential for the intervention provider to take the time to build a warm and trusting relationship with the parents. They believe that such a program must be flexible and that the program provider must adapt to the parents they meet on a case-by-case basis. Parents shared the same view regarding the program provider’s attitude. This moment of initial contact is essential to enable them to reveal themselves. Most parents also stressed the importance of this preliminary step before the intervention begins to promote contact:

P5: “Parents will definitely need much support and perhaps one or two meetings to allow them to open up with a trained professional that can listen to them and guide them.”

In summary, the interviewed parents highlighted the following program benefits: (1) the relevance of the program’s main target, (2) the complementary of the intervention’s individual and couple components, (3) the training in taking back control over the long term which can be used beyond the cancer context, (4) the relevance of the target audience selection (distressed parents including single-parent families), (5) the program’s manualized aspect (in particular by proposing a manual to parents), and (6) the program provider’s empathetic attitude. According to healthcare professionals, reported drawbacks included (1) the intervention program could be an overload for parents (e.g., completion of homework), (2) the wording of the parent manual is considered too long and complicated in its writing style, (3) sessions at the parents’ homes are not always feasible. In this case, some adjustments will be necessary.

### Suggestions for program improvement

#### Suggestions for improving manuals

One healthcare professional (H1) suggested improving the provider manual by simplifying the technical writing style so that it is accessible to various healthcare professionals (doctors, nurses, social workers).

With regard to the parent’s manual, all of the professionals and two mothers (P4, P6) suggested simplifying and reducing the texts. The document should contain fewer pages and more illustrations. It was also suggested to avoid using terms that are reminiscent of school in this manual, for example, the word “homework.” Some professionals (H1, H3, H4) stressed that the language used must be accessible to everyone even if their level of literacy or proficiency in French is weak. The style should therefore be simplified and the text “written at a grade 4 level” (H1). Additionally, although two couples (P1, P2) (P3, P4) judged the writing style to be simple, they were able to see how difficult it could be for other parents and also suggested simplifying the document. Two professionals (H2, H4) also indicated that it would be better to offer a 4-page double-sided booklet rather than a manual that may be too complex and confusing for parents. One professional (H1) suggested replacing the manual with documents in a video format. Finally, all healthcare professionals and one couple (P3, P4) proposed to make the manual more attractive for parents to facilitate their adherence to the program. They suggested a shorter, more fun and simple manual, going straight to the point, and using codes they already know:

P4: “You know...like in a comic book it is always caricatural, and so at one point when you are reading, it makes you smile, it makes you feel good, you can relax...”

#### Suggestions for improving toolkits and illustrations

Professionals and parents provided suggestions for improving worksheets. One healthcare professional (H1) emphasized the importance of allowing more freedom by avoiding the ranking of solutions: “we could put more emphasis on the generation of ideas before evaluating them.” More specifically, it would mean letting parents brainstorm solutions as they are asked to do in the process of selecting problems to solve. On the other hand, one couple (P3, P4) suggested that these leaflets also include codes that are already familiar in pediatrics:

P4: “You know in oncology; the nurse gives me tools with smiley faces and emoticons that speaks to me.”

Healthcare professionals and parents suggested improving illustration. First, healthcare professionals expressed reservations about the choice of the canoe trip to illustrate the use of PSST; this metaphor having little impact because “there are few people who go on canoe trips” (H1). They therefore suggested that it is necessary to ensure adequate cultural adaptation by mobilizing more familiar concepts such as mountains or other concepts:

H2: “For the canoe metaphor, travel is good, but if you’re dealing with a variety of multicultural realities, the canoe will not mean anything, and maybe variants could help...like cars, horses, etc.”

Second, one healthcare professional (H4) and one mother (P2) recommended proposing other case studies or a larger variety of cases to create “an image bank.” However, the other healthcare professionals argued that the case study is quite broad and representative of the clinical reality of many parents. It is therefore considered more useful not to integrate case studies in the program documents and to instead offer them alternative examples during sessions in a personalized way, i.e., in keeping with the lived experience of the parent encountered. In fact, one mother (P2) explained that she “finds it helpful to read examples of plausible situations.”

In summary, professionals and parents offered suggestions on improving textbooks, tools and program illustrations. They recommended simplifying the writing of both manuals, shortening, and making them more appealing with illustrations, specifically for the parent manual. Regarding the worksheets, they recommended allowing more freedom to generate solutions without imposing a hierarchization immediately and using codes known by patients as those proposed on medical records in pediatric oncology. As for illustrations, they suggested more clinical cases and better adaptation to the patient’s culture. The Table 2 summarizes the evaluations and the modifications made to the intervention program.

**Table 2:** Summary of the evaluations and modifications made to the program and its tools

Theme	Benefits	Limitations and suggestions for improvement	Suggestions for modifications
1—Program relevance and acceptability			
Emotional support	Aim of the program: psychological and emotional support for parents		
Regain control	Improve parental capacity in the management of everyday practical difficulties		
Target audience	According to parents: couples and single-parent families are targets consistent with the nature of the difficulties encountered in pediatric oncology	According to healthcare professionals: it is possible that the program may not be adaptable to the reality of some parents (overload)	The program will be more adaptable in order to respect the parents’ reality: flexibility regarding appointment times, session location, and “homework” requirements
2—Program material			
Provider manual	The manualized aspect is appreciated	Terminology is too reminiscent of school (e.g., homework) Avoid using technical terms so that it is accessible to non-psychologists	Modify the school-like terms (“homework” replaced with “tasks to complete at home”) and techniques in the manual (e.g., operationalization of the problem replaced with characteristic of the problem)
Parent manual	Helps expand learning and knowledge of the program outside of sessions	According to healthcare professionals: too complex, too extensive, and the general level of writing style is too elevated for some parents: Simplify writing (aim for grade 4 level), reduce the amount of text and present it in a four-page booklet, remove the terms that are too complicated, and add illustrations to make it more attractive	Rewrite in a simpler way and create a computerized version and video capsules illustrating the program sessions. The print format will be smaller and include illustrations.
Worksheets	Helps maintain practice outside of sessions	Give parents more freedom in their selection of solutions to their problem, avoid suggesting ranking solutions right away, and add illustrations and visual codes that are already familiar in pediatrics (emoticons)	The worksheets will be more attractive. We will add images, change the font of the text and titles, and modify the layout. The “generate solutions” worksheet will no longer suggest ranking parents’ responses
Illustrations and case study	Helpful, they relate to examples of plausible situations	Consider the cultural aspect in the selection of metaphors	The professionals will be asked to adapt the canoe metaphor and case studies to the cultural background of the participating parents
3—Practical implementation			
Location of the intervention	Parents approve of the flexibility in being able to choose the location of the intervention (at the hospital or at home)		
Time of the intervention	Flexibility in the choice of the time of the first meeting (approximately 4 weeks after the diagnosis)		
Number of sessions	Some parents think the number of sessions is sufficient	For healthcare professionals, some parents may give up because the duration (6 sessions) will be considered too long	The program will be more flexible in terms of the number of sessions. It will be possible to complete the 4 individual sessions only, if the parents prefer
Tasks to complete at home	Tasks can be beneficial if they are not imposed	Prescription of tasks to complete at home may be too demanding for parents	Prescription of tasks to complete at home will be more flexible and tasks will be suggested, not imposed on parents
4—Program procedures			
Individual sessions	Problem-solving training sessions serve the purpose of regaining control and facilitating emotional expression		
Couple sessions	Sessions promote marital communication and the ability to work as a team		
5—Program provider’s attitude^1^	It is important and appreciated that a time to build the relationship be planned in the program		

^1^This theme was not initially part of the questionnaire. It was addressed by participants during interviews

## Discussion

In line with recommendations on program development, we refined the design of TAKING BACK CONTROL TOGETHER, a manualized intervention program to reduce parental distress in pediatric oncology [20, 24]. Following data collection, responses to questionnaires and focus groups or interviews with professionals and parents, we identified strengths and limitations, and collected suggestions for modifications of the intervention.

The results support the pertinence of the intervention. The concept of the program and its components aim to restore control through individual sessions that offer PSST and maintain unity within parents’ couples by reinforcing dyadic coping and marital communication during the couple sessions. The results also highlighted benefits such as offering emotional support, and an opportunity to take back long-term control, i.e., well beyond the oncological treatments. The benefits also concerned the relevance of offering support for parents with high levels of distress while including single-parent families; and the program’s manualized aspect, which promotes its transmission to healthcare professionals and parents alike. These aspects were considered relevant by participants. They are also consistent with recommendations about psychological interventions in the area of pediatric cancer [37, 38]. Participants highlighted some limitations, including the fact that this intervention could be an additional burden for some, that the wording of the parent manual used a writing style difficult to understand, and was probably too detailed. Participants also questioned the feasibility of meeting parents at home for the couple sessions. Although these limitations did not challenge the acceptability and relevance of the program, they could be barriers to the participation of some highly distressed parents.

To address these limitations, modifications were made with respect to the comments and improvement tips provided by the participants. Firstly, the writing style of both provider and parent manuals have been simplified to provide the reader with a better understanding. We removed technical language from the provider manual to make it more accessible for all professionals including nurses, social workers, or counselors. The parent manual text was simplified in its writing to make it more understandable for a wide range of people in Canada whose mother tongue is not French. Consistent with the fact that training in therapeutic procedures requires a therapeutic relationship and active participation, distinct from a teacher-student relationship [14, 27], we removed school-like terms. The parent manual was also simplified, and more illustrations were integrated. The program’s video clips are being recorded in order to propose an electronic version of the manual accessible through a dedicated internet platform, in coherence with other manualized programs that offer computerized versions in the same field [39–41]. Secondly, the “generating solutions” worksheet no longer requires direct ranking from parents. This is required after the generation of solutions. In line with the spirit of PSST [27], parents can thus search for solutions more freely, without there being a risk of self-censorship linked to a premature evaluation of the feasibility of this type of solution. In fact, PSST including the search for solutions requires the patient to stop and think freely [14, 27]. Thirdly, the canoe trip metaphor and case studies are being adapted to the parents’ cultural background. In order to promote a connection with the illustrations made in the program, it is indeed useful to propose contexts that are close to the parents’ experiences. This way, we are aligning ourselves with the intercultural psychological interventions field, which supports the effectiveness of adapting therapeutic discourse to the patient’s culture [42]. Fourthly, following comments from participants to decrease the burden related to the intervention, we decided that the place and time for delivery will be chosen by participants themselves. Hence, meetings can take place in a home or hospital setting. Notably, the program is focused on alleviating overload; thus, delivery mode should remain coherent with this concept [20]. In addition, this more flexible framework fits better with the reality of psychological intervention practice in the day-to-day care of childhood cancer [39].

Beyond the results themselves, we wish to highlight some methodological aspects of this study. The mixed methodology allowed us to generate a refined evaluation of the program and detail elements of strength and weaknesses. Using a brief survey at the start helped highlight the core topics to be addressed in individual and group interviews, thus allotting as much time as necessary to what seemed most important to the participants in the qualitative data collection. The qualitative data helped expand these responses by providing detailed justifications about opinions and examples. Moreover, collecting complementary points of view from both parents and professionals in separate meetings also helped enrich data by limiting the risk of self-censorship. Indeed, some comments including criticisms or worries clearly indicated that parents might have felt intimidated to speak openly about their relation to the clinic in the presence of the professionals, while professionals would probably have been more guarded not to hurt or offend parents if groups had been mixed. The qualitative dimension was not intended to confirm the statistical validity of the opinions expressed but rather to reach a level of depth, precision, and optimal reliability to guide changes and improvements to be made to the program.

We must acknowledge some limitations of this study. Parents interviewed in this study were from the province of Quebec, Canada and spoke French. Although we had some diversity among professionals, it was not possible in parents, for feasibility purposes. It is possible that additional culturally anchored elements would have been identified as weaknesses if parents’ background had been more varied. We used social media networks to invite parents, which limits the participation to people who use this technology. We were also faced with the impossibility of assembling all parents in one focus group as originally planned in our study. However, we conducted individual interviews that led to a diversity of pertinent experiences.

## Conclusions

In conclusion, this study is the second step in defining a support program designed for parents confronted with pediatric cancer, TAKING BACK CONTROL TOGETHER. Having defined the program concept, we submitted it to end-users to assess its future relevance and acceptability, and improve the delivery mode and its tools/activities. Collecting information from end-users enabled us to engage in improvements to the program. The current version is ready to be pilot-tested. The future pilot will be focused on evaluating treatment fidelity and overall feasibility in a pre-post study conducted with parents whose child recently received a pediatric cancer diagnosis. It will also measure the change associated with the intervention.

## Abbreviations

CBT: Cognitive behavioral therapy
PSST: Problem-solving skills training
